# Supplementary material for: Consumption of single products versus fixed-dose combination medicines for hypertension and hyperlipidemia during 2015–2019 in South Korea
Source: PLoS One. 2021 Dec 15;16(12):e0259467. doi: 10.1371/journal.pone.0259467 (PMC8673596; doi:10.1371/journal.pone.0259467)
Supplement: Attachment — Submitted filename: Revision manuscript PlosOne.docx [file pone.0259467.s001.docx]

The manuscript is well written and clear to the reader.

Only two remarks:

- In "Use by drug classification" chapter, paragraph 5, line 2, after "...carvedilol (4.8 DIDs, 67 million USD", it needs a parenthesis ")";
- In "Discussion" chapter, paragraph 1, line 9, it is "...followed by C08 (CCB), with 70.7 DIDs".
