# Supplementary material for: Consumption of single products versus fixed-dose combination medicines for hypertension and hyperlipidemia during 2015–2019 in South Korea
Source: PLoS One. 2021 Dec 15;16(12):e0259467. doi: 10.1371/journal.pone.0259467 (PMC8673596; doi:10.1371/journal.pone.0259467)
Supplement: Attachment — Submitted filename: Response to reviewer.docx [file pone.0259467.s002.docx]

**Consumption of single products versus fixed-dose combination medicines for hypertension and hyperlipidemia during 2015-2019 in South Korea**

Response to reviews’ comments

Dear Masaki Mogi

We appreciate the opportunity to revise our manuscript (manuscript ID PONE-D-21-21296), based on your comments in an e-mail that was dated August 13, 2021. The manuscript has been revised based on the comments that were provided.

We have enclosed the revised manuscript with the changes highlighted as well as a response to the reviewers comment (below). Also, we revised the reference list.

Thank you for your ongoing consideration, and we look forward to hearing from you.

Sincerely,

Dong-Sook Kim

**Response to the reviewer #1’s comment**

We would like to thank the reviewer for improving the manuscript.

The manuscript is well written and clear to the reader.

Only two remarks:

In "Use by drug classification" chapter, paragraph 5, line 2, after "...carvedilol (4.8 DIDs, 67 million USD", it needs a parenthesis ")";

In "Discussion" chapter, paragraph 1, line 9, it is "...followed by C08 (CCB), with 70.7 DIDs".

**Author’s Response**: Thank you for the time and effort you invested in thoroughly reviewing this manuscript. According to your comment, we revised.

**Response to the reviewer #2’s comment**

We would like to thank the reviewer for improving the manuscript.

The present study is a retrospective study based on the Korean national health insurance claims database, describing the trends in consumption of fixed-dose combinations of lipid-lowering and antihypertensive drugs. The study is of interest and the following comments should be taken into account to improve its quality.

-The available data regarding triple combinations (e.g. 2 antihypertensive drugs+statin or 1 antihypertensive drug+statin+ezetimibe) should be reported.

**Author’s Response**: Thank you for your comment.

The unit of analysis of this study was to medicine. We analyzed the characteristics of patients who use fixed dose combination (FDC). However, multiple use of hypertension and hyperlipidemia combinations in the patient unit was not analyzed.

-The results of large meta-analyses in the field should be discussed.

-Pharmacokinetic/pharmacodynamic data regarding potential interactions should be elaborated in the Discussion section.

**Author’s Response**: Thank you for these helpful comments. However pharmaceutical company must submit approval data to Food and Drug Administration, so doctors might prefer to prescribe fixed dose combination (FDC) of hypertensive drugs or hyperlipidemia rather than to combine each drug. Therefore, we did not consider regarding potential interactions because this study examined the pattern or trend of combination drugs. Also, we mentioned the large meta-analyses of fixed-dose combination drugs and changed the Discussion section as follows:

According to several meta-analysis studies, the FDC treatment is associated with a significant improvement in adherence and persistence in comparison with single therapy or FECs, thereby increase of BP control effect, so these strategies have been preferred compared with conventional therapies.

-Figure 1, despite being comprehensive, is complicated. An additional figure is suggested showing the consumption trends of the most common antihypertensive drugs and combinations, presenting the name of the class (e.g. Diuretics) and not the drug codes in order to be easily interpretable by clinicians.

**Author’s Response**: Thank you for your suggestion. According to your comment, we revised.
